# Long Island College Hospital

**Published:** 1860-10

**Authors:** 


### Long Island College Hospital.

Among our advertisements will be found that of the above Institution, for the next Preliminary and Regular Courses of Lectures. It will be seen that in addition to the Chair of "Principles and Practice of Surgery," that of "Operative Surgery and Surgical Anatomy," under the direction of Joseph C. Hutchison, M. D., is inserted in the curriculum of studies, as a distinct branch of instruction in Surgery, making eight regular Chairs in the College.

With such a catalogue of talented Professors, headed by the name of Dr. Austin Flint, in the Chair of Practical Medicine and Pathology, we confidently predict a successful future for the College. In Practical Diagnosis of diseases of the chest, by Dr. Flint, from physical signs, we suspect the student will find a treat.

With the means at their command to make practical demonstrations in Clinical Medicine, from the connection of the College with an extensive Hospital, we doubt not the daily lectures of the Faculty, in this department, will be highly instructive and entertaining.

We commend the Institution to Medical students going North, with every confidence in the ability of the Faculty and in their abundant facilities for teaching in every department of Medical science.

